# The impact of transcriptional tuning on *in vitro* integrated rRNA transcription and ribosome construction

**DOI:** 10.1093/nar/gku307

**Published:** 2014-05-03

**Authors:** Brian R. Fritz, Michael C. Jewett

**Affiliations:** 1 Department of Chemical and Biological Engineering, Northwestern University, 2145 Sheridan Road, Evanston, IL 60208, USA; 2 Interdepartmental Program in Biological Sciences, Northwestern University, 2205 Tech Drive, Evanston, IL 60208, USA; 3 Northwestern Institute on Complex Systems, Northwestern University, 600 Foster Street, Evanston, IL 60208, USA; 4 Institute for Bionanotechnology in Medicine, Northwestern University, 303 E. Superior, Chicago, IL 60611, USA; 5 Chemistry of Life Processes Institute, Northwestern University, 2170 Campus Drive, Evanston, IL 60208, USA

## Abstract

*In vitro* ribosome construction could enable studies of ribosome assembly and function, provide a route toward constructing minimal cells for synthetic biology, and permit the construction of ribosome variants with new functions. Toward these long-term goals, we recently reported on an integrated, one-pot ribosomal RNA synthesis (rRNA), ribosome assembly, and translation technology (termed iSAT) for the construction of *Escherichia coli* ribosomes in crude ribosome-free S150 extracts. Here, we aimed to improve the activity of iSAT through transcriptional tuning. Specifically, we increased transcriptional efficiency through 3′ modifications to the rRNA gene sequences, optimized plasmid and polymerase concentrations, and demonstrated the use of a T7-promoted rRNA operon for stoichiometrically balanced rRNA synthesis and native rRNA processing. Our modifications produced a 45-fold improvement in iSAT protein synthesis activity, enabling synthesis of 429 ± 15 nmol/l green fluorescent protein in 6 h batch reactions. Further, we show that the translational activity of ribosomes purified from iSAT reactions is about 20% the activity of native ribosomes purified directly from *E. coli* cells. Looking forward, we believe iSAT will enable unique studies to unravel the systems biology of ribosome biogenesis and open the way to new methods for making and studying ribosomal variants.

## INTRODUCTION

*Escherichia coli* 70S ribosomes are complex macromolecular machines consisting of three ribosomal RNA (rRNA) molecules and 54 ribosomal proteins (r-proteins). The large subunit, 50S, consists of 23S and 5S rRNA and 33 r-proteins, while the small subunit, 30S, consists of 16S rRNA and 21 r-proteins. 70S ribosomes are capable of sequence-defined polymerization of amino acid monomers into proteins.

Over the last several decades, *in vitro* reconstitution of ribosomes from purified native rRNA and r-proteins have played a transformative role in dissecting molecular mechanisms that define ribosome assembly, including r-protein maps (1–6). However, assembly of *E. coli* ribosomes from *in vitro* transcribed rRNA using classical reconstitution methods remains inefficient, especially for the 50S subunit. Assembly of 50S subunits using *in vitro* transcribed 23S rRNA have led to only ‘marginally functional’ particles (7). Inefficiencies arise because *in vitro* transcribed 23S rRNA lacks the appropriate post-transcriptional modifications, as elegantly shown by Green and Noller (8). Even *in vivo* 50S assembly can be disrupted by a lack of post-transcriptional methylation of 23S rRNA (9). It is further hypothesized that the separation of 23S rRNA transcription and ribosome assembly used in classical 50S reconstitution experiments may also reduce assembly efficiency (10,11).

Despite inefficiencies of classical reconstitution methods, *in vitro* construction of *E. coli* ribosomes is a topic of rapidly growing interest. The driving force behind this growth is 3-fold. First, new approaches in cytoplasmic mimicry *in vitro* have enabled more active and efficient ribosome assembly and cell-free protein synthesis (CFPS) systems (11–13). Second, there is resurgence in efforts to build minimal cells from defined components (14–18). Third, the lack of cellular viability constraints opens the aperture to study rRNA variants with deleterious miscoding phenotypes or altered functions that would not be identified in classical genetic screens (7). For example, *in vitro* systems should make possible the study of the protein synthesis machinery under a variety of non-physiological conditions, such as altered pH, temperature and redox level (8,19–25). Additionally, *in vitro* systems have greater potential than *in vivo* systems to explore ribosomal variants (7). Specialized orthogonal ribosomes, which utilize 16S rRNA with a modified anti-Shine Dalgarno sequence, offer an alternative approach to create and study independent pools of 30S subunits *in vivo* (26). However, this technique is limited to 30S subunits because 50S subunits freely exchange between pools of native and orthogonal 30S subunits (27). Further, leaky translation of mRNAs by orthogonal ribosomes can lead to dominant growth defects that also limit this approach (26). *In vitro* ribosome construction could overcome these limitations and allow for manipulation of both ribosomal subunits.

**Figure 1.**
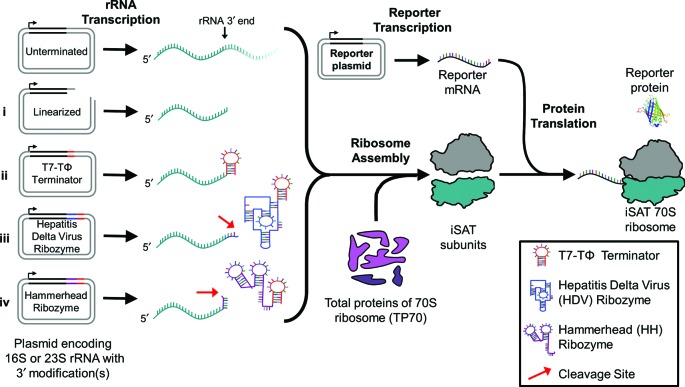
Integrated 16S and 23S rRNA synthesis, 70S ribosome assembly, and reporter protein translation (iSAT) using rRNA constructs containing 3′ gene modifications. rRNA are transcribed from plasmids by T7 RNA polymerase and assembled with purified total protein of the 70S ribosome (TP70) into 70S iSAT ribosomes. Newly assembled ribosomes translate co-transcribed mRNA encoding the reporter proteins luciferase or sfGFP. These steps occur simultaneously at 37°C, and active luciferase or sfGFP synthesis is used as a measure of ribosome activity. ‘Unterminated’ refers to the use of plasmids pWK1 and pCW1 encoding 16S rRNA and 23S rRNA, respectively, as previously reported (11). Modifications to the 3′ ends of the rRNA genes include (i) 3′ linearization by digestion with the restriction enzyme Bsu36I (pWK1) or Aflll (pCW1) to allow for run-off transcription, or addition of (ii) T7-TΦ terminators, (iii) hepatitis delta virus (HDV) ribozymes or (iv) hammerhead (HH) ribozymes. Ribozymes are expected to self-cleave, denoted by red arrows, to minimize the number of additional bases included beyond native 16S or 23S rRNA 3′ ends. T7 terminators are included after ribozyme genes to limit excess transcription.

Recently, our lab developed an integrated synthesis, assembly and translation (iSAT) technology for *E. coli* 70S ribosome biogenesis *in vitro* (11). iSAT combines *in vitro* rRNA transcription, ribosome assembly of the rRNA with purified total protein of 70S ribosomes (TP70), and translation of a reporter protein as a measure of ribosome activity (Figure 1) (11). This contrasts with previous approaches of *in vitro* ribosome reconstitution because rRNA transcription and ribosome assembly are co-activated in one reaction as it occurs *in vivo* (8,22–23,25). Further, ribosome assembly and functional activity (i.e. translation) are linked. The key to this methodology lies in the recreation of near-physiological salt conditions to allow these biological processes to occur at 37°C without magnesium or temperature shifts previously required for ribosome reconstitution from purified components (11,22–23,28).

Unfortunately, iSAT remains inefficient. 70S iSAT ribosomes using *in vitro* transcribed rRNA showed 12% luciferase synthesis activity compared to ribosomes assembled in the same system from purified total rRNA of 70S ribosomes (TR70) and TP70 (11). For comparison, in conventional reconstitution systems, 50S subunits assembled from *in vitro* transcribed *E. coli* 23S rRNA have about 0.02% activity compared to 50S subunits assembled from purified native 23S rRNA as measured by the fragment reaction, and the addition of the osmolytes telithromycin and trimethylamine-oxide only increases activity to 3% (25). As a result of these inefficiencies, our development of the iSAT system originally focused on individual subunit assembly, as opposed to simultaneous assembly of both subunits, in order to improve reporter signal strength and identify differences between 30S and 50S subunit assembly.

Here we hypothesized that iSAT activity could be increased by transcriptional tuning to improve rRNA transcription and processing efficiency. First, we introduced 3′ gene modifications to existing rRNA-encoding plasmids. Previously, the plasmids encoding 16S and 23S rRNA lacked transcriptional termination and likely 3′ end processing, resulting in rRNA of variable length. We tested modifications including plasmid linearization, transcriptional termination and ribozyme cleavage (Figure 1). Second, we manipulated plasmid and polymerase concentrations. With co-transcription of three plasmids encoding 16S and 23S rRNA and a reporter protein mRNA, all utilizing the T7 RNA polymerase (RNAP), we hypothesized that improving transcriptional balance would be critical to improving iSAT activity. Third, in an attempt to better mimic *in vivo* ribosome biogenesis, we used a native rRNA operon in the iSAT system to achieve stoichiometrically balanced synthesis of all three rRNA molecules and to utilize native RNase processing. For the operon-based iSAT system, concentrations of transcriptional components were optimized as with the original separate plasmid iSAT system. Throughout this research, changes to the iSAT system were assessed via measurement of reporter protein synthesis and visualization of rRNA by gel electrophoresis. The efforts reported here achieved a 45-fold overall improvement in iSAT protein synthesis activity, and demonstrated that RNA processing enzymes are present and active in the S150 extract. We anticipate that this newfound understanding and technical advance will lead to new efforts in studying and manipulating ribosome biogenesis.

## MATERIALS AND METHODS

### 70S Ribosome purification

For native *E. coli* 70S ribosome purification, MRE600 *E. coli* cells were grown to 3.0 OD_600_ in a 10 l fermentor (Sartorius), pelleted, and flash-frozen. Cell pellets were resuspended in Buffer A (20 mM Tris-HCl (pH 7.2 at 4°C), 100 mM NH_4_Cl, 10 mM MgCl_2_, 0.5 mM EDTA, 2 mM DTT) at a ratio of 5 ml of buffer per 1 g of cells. 200 μl Halt Protease Inhibitor Cocktail (Thermo Fisher Scientific Inc.) and 75 μl RNase Inhibitor (Qiagen) were added for every 4 g of cells in the suspension. The cells were lysed at approximately 20 000 psi with an EmulsiFlex-C3 homogenizer (Avestin). An equivalent dose of RNase Inhibitor and 3 μl 1 M DTT per milliliter were added to the lysate prior to two clarification spins at 30 000 *g* and 4°C for 30 min. Supernatant equivalent to S30 crude extract was recovered and gently layered into Ti45 ultracentrifuge tubes on top of an equivalent volume of a sucrose cushion, Buffer B (20 mM Tris-HCl (pH 7.2 at 4°C), 100 mM NH_4_Cl, 10 mM MgCl_2_, 0.5 mM EDTA, 2 mM DTT, 37.7% sucrose). Samples were then centrifuged at 90 000 *g* (33 900 rpm in Ti45 rotor) and 4°C for 20 h. Supernatant was recovered for S150 extract (see below), and the remaining clear ribosome pellet was gently washed and resuspended in Buffer C (10 mM Tris-OAc (pH 7.5 at 4°C), 60 mM NH_4_Cl, 7.5 mM Mg(OAc)_2_, 0.5 mM EDTA, 2 mM DTT). Concentration of resuspended ribosomes was determined from A_260_ NanoDrop readings (1 A_260_ unit of 70S = 24 pmol 70S (29)). Ribosomes were then aliquoted and flash-frozen for use as purified 70S ribosomes and for purification of native rRNA and r-proteins.

### S150 extract preparation

Supernatants collected from the 70S ribosome pellets were spun at 150 000 *g* and 4°C for an additional 3 h. The top two-thirds of the supernatant was recovered and dialyzed in reconstituted Spectra/Por^®^ 3 dialysis membrane tubing (3500 Da MWCO) against a high salt S150 extract buffer (10 mM Tris-OAc (pH 7.5 at 4°C), 10 mM Mg(OAc)_2_, 20 mM NH_4_OAc, 30 mM KOAc, 200 mM KGlu, 1 mM spermidine, 1 mM putrescine, 1 mM DTT). Dialysis buffer volume was 50-fold greater than sample volume and exchanged after 2 h for three dialysis steps. A fourth dialysis was performed overnight for 15 h. Extract was clarified at 4000 *g* for 10 min and concentrated 6- to 8-fold in 3000 Da MWCO Centriprep concentrators (EMD Millipore) to account for dilution during preparation. Final protein concentration of S150 extract was 4.6 ± 0.5 mg/ml, as determined by a Bradford assay using bovine serum albumin as a standard. S150 extract samples were then aliquoted, flash-frozen and stored at −80°C.

### Total protein of 70S ribosomes (TP70) preparation

Purified *E. coli* 70S ribosomes were diluted 5-fold in Buffer C and passed through a second sucrose cushion by ultracentrifugation as in the initial ribosome purification. The resulting pellet was again resuspended in Buffer C and the polyamines spermine and spermidine were added to final concentrations of 0.2 mM and 2 mM, respectively. One-tenth of the sample volume of 1 M Mg(OAc)_2_ was added, and two volumes of glacial acetic acid were added to precipitate rRNA. This sample was vortexed at 4°C for 45 min and then centrifuged at 16 000 *g* for 30 min to pellet precipitated rRNA. Supernatant containing r-proteins was collected and mixed with 5 volumes of chilled acetone and stored overnight at −20°C. Precipitated protein was then collected by centrifugation at 10 000 *g* for 30 min, dried and resuspended in simplified high salt buffer with urea (10 mM Tris-OAc (pH = 7.5 at 4°C), 10 mM Mg(OAc)_2_, 200 mM KGlu, 1 mM DTT, 6 M urea (buffer was mixed with 1 g/l bentonite for 1 h at 4°C and bentonite was filtered out (0.2 μm) prior to use)). The sample was transferred to midi-size 1000 Da MWCO Tube-O-Dialyzers (G-Biosciences) and dialyzed overnight against 100 volumes of simplified high salt buffer with urea. The sample was then dialyzed against 100 volumes of simplified high salt buffer without urea three times for 90 min each. This sample was clarified at 4000 *g* for 10 min and concentration was determined from A_230_ NanoDrop readings (1 A_230_ unit of TP70 = 240 pmol TP70 (29)). TP70 samples were then aliquoted, flash-frozen and stored at −80°C.

### Total RNA of 70S ribosomes (TR70) preparation

Purified *E. coli* 70S ribosomes were diluted below 250 A_260_/ml with Buffer C and mixed with 0.1 volume 10% w/v SDS, 0.05 volume 2% w/v bentonite and 1.0 volume 70% v/v phenol. The sample was vortexed for 8 min at 4°C, then centrifuged at 12 500 *g* for 15 min. The aqueous phase was collected, mixed with 1.0 volume 70% v/v phenol, shaken for 5 min at 4°C, centrifuged at 12 500 *g* for 15 min and collected again. 2 volumes of chilled ethanol were added, and the sample was stored at −20°C overnight to precipitate rRNA. Precipitant was collected by centrifugation at 15 000 *g* for 45 min, washed with 0.5 volumes ethanol and dried. TR70 pellets were then resuspended in Buffer J (10 mM Tris-OAc (pH = 7.5 at 4°C) and 7.5 mM Mg(OAc)_2_) and concentration was determined from A_260_ NanoDrop readings (1 A_260_ unit of TR70 = 24 pmol TR70 (29)). TR70 samples were then aliquoted, flash-frozen and stored at −80°C.

### T7 RNA polymerase preparation

T7 RNAP was prepared as previously described (30). Then, T7 RNAP was dialyzed in a midi-size 1000 Da MWCO Tube-O-Dialyzer overnight at 4°C, against 100 volumes of the same simplified high salt buffer without urea used for TP70 preparation (see above). T7 RNAP was then concentrated in 3000 Da MWCO Centriprep concentrators by spinning at 10 000 *g* for 15–45 min intervals. T7 RNAP was concentrated to 1.5 mg/ml, as determined by Bradford assay using BSA standards.

### Plasmid constructions

Primers used for plasmid constructions are listed in Supplementary Table S1. 3′ modifications to rRNA-encoding plasmids pWK1 (16S rRNA) (31) or pCW1 (23S rRNA) (32) were introduced through inverse polymerase chain reaction (PCR) with phosphorylated primers and blunt end ligation of the linear product. Upon transformation and plasmid purification, the resulting constructs were DNA sequenced by the Northwestern University Genomics Core to confirm proper modifications. For constructs including ribozymes, the terminated constructs p16S-T and p23S-T were first created and the ribozyme sequences were inserted between the rRNA gene and terminator sequence using a similar method. Likewise, inverse PCR and blunt end ligation was used for insertion of the T7 promoter sequence into pAM552A, a derivative of the pLK35 plasmid encoding the *rrnB* operon (33), to create pT7rrnB. The A2058U clindamycin resistance mutation of 23S rRNA was introduced into the 23S rRNA gene sequence of pT7rrnB as previously reported (11).

### iSAT and cell-free transcription and translation (TX-TL) reactions

Cell-free reactions were set up as previously described (11). Reagents are listed in Supplementary Table S2 showing concentration ranges used for optimizations. Reagents were premixed and added to S150 extract with purified ribosomal components (TP70, TR70 or 70S ribosomes) to a final volume of 15 μl. Tubes were then incubated at 37°C. The final optimized reaction conditions for the separate plasmid and operon-based iSAT systems are also shown in Supplementary Table S2.

### iSAT ribosome purification

Twenty-four 15 μl operon-based iSAT reactions were incubated at 37°C for 3 h, placed on ice, pooled and diluted with 2 volumes of chilled Buffer A. Pooled iSAT reactions were then loaded into Ti70 ultracentrifuge tubes containing a 13 ml sucrose cushion of Buffer B with 10 ml of Buffer A layered on top. After loading the diluted iSAT reactions, tubes were filled to ∼26 ml with Buffer A, balanced, and spun at 90 000 *g* (35 000 rpm in Ti70 rotor) and 4°C for 18 h. Upon completion, supernatant was removed and the clear ribosome pellet was resuspended in a HEPES-based high salt buffer (50 mM HEPES-KOH (pH = 7.6 at room temperature), 10 mM MgGlu, 200 mM KGlu, 0.5 mM EDTA, 1 mM spermidine, 1 mM putrescine, 2 mM DTT). The concentration of purified iSAT ribosomes was determined from A_260_ NanoDrop readings (1 A_260_ unit of 70S = 24 pmol 70S (29)). To assess the activity of iSAT-assembled ribosomes, purified iSAT ribosomes were then used at reaction concentrations of 100 nM in S150 extract-based cell-free TX-TL reactions as described above.

### Luciferase quantification

When producing luciferase as a reporter protein from the plasmid pK7Luc, 15 μl iSAT reactions were performed in 1.5 ml microtubes and incubated in heat blocks within an incubator for 4 h. Microtubes were then placed on ice to stop the reactions. Luciferase concentration in each reaction was determined by mixing 1–10 μl of sample with 30 μl ONE-Glo^TM^ Luciferase Assay System (Promega) in a white half-area 96-well plate. Resulting luminescence was read at 26°C in a BioTek Synergy2 plate reader over 20 min. The maximum relative luminescence units were converted to molar concentrations using a standard curve generated from a dilution series of QuantiLum® recombinant luciferase (Promega). To control for background expression, reactions were performed without additional rRNA or rRNA genes and assessed for active luciferase production, and this value (∼1 nmol/l at 4 h) was subtracted from experimental expression values.

### sfGFP quantification

When producing superfolder GFP (sfGFP) as a reporter protein from the plasmid pY71sfGFP, 15 μl iSAT reactions were performed in flat-capped PCR tubes and incubated in a CFX96^TM^ real-time thermal cycler (Bio-Rad). sfGFP production was monitored by measuring fluorescence at 5 or 30 min intervals (excitation: 450–490 nm, emission: 510–530 nm). Relative fluorescence units were converted to molar concentrations using a standard curve generated from a dilution series of purified recombinant sfGFP. As with luciferase, control reactions were performed without additional rRNA or rRNA genes and assessed for active sfGFP production, and this value (∼15 nmol/l at 6 h) was subtracted from experimental expression values.

### RNA denaturing agarose gel electrophoresis

Agarose gels were prepared with 1.0% agarose, 2.2 M formaldehyde, 1× MOPS buffer (20 mM MOPS, 2 mM NaOAc, 1 mM EDTA, adjusted to pH 7.0 with NaOH) and 1× GelRed^TM^ dye (Biotium). Samples were prepared by RNA purification of standard iSAT reactions without reporter plasmid using Bio-Rad's Aurum^TM^ Total RNA Mini kit. The kit's bacteria protocol was followed with the exception of initial lysozyme treatment, as no cell lysis was required. Controls included purified rRNA from subunits or ribosomes and prepared as previously reported (11). Ladder was 0.5–10 kb RNA ladder from Life Technologies. Samples, ladders and controls were denatured in 1× blue loading dye (New England BioLabs), 1× MOPS buffer, 40% formamide and 8% formaldehyde at 70°C for 10 min, and then placed on ice for 5 min. Gels were pre-run at 100 V for 10 min. Gels were then loaded with RNA and run at 50 V for 3 h. Upon completion, gels were imaged in a Bio-Rad Gel Doc^TM^ XR+ station. Images were inverted and contrast was adjusted to improve band visibility, and band intensities were approximated with Image Lab™ software.

## RESULTS

### Optimization of iSAT reaction conditions

As previously described, 70S iSAT reactions consist of transcription of 16S and 23S rRNA, assembly of rRNA with purified TP70, and measurement of ribosome activity by transcription and translation of a reporter gene, either luciferase or superfolder GFP (sfGFP) (Figure 1). These processes occur simultaneously at 37°C in 15 μl reactions. The reactions consist of crude ribosome-free S150 *E. coli* extract containing cytoplasmic translation and assembly factors, and salts, buffers and substrates necessary for transcription and translation (Supplementary Table S1) (11,12).

The goal of this study was to improve iSAT through transcriptional tuning of the rRNA. First, however, we hypothesized that increasing the concentration of the S150 extract components could improve iSAT activity. Previous iSAT work used dilute S150 cell extract (∼1 mg *E. coli* protein per milliliter reaction) (11), which is ∼200- to 300-fold less than typical *E. coli* cytoplasmic protein concentration (34). Since ribosome assembly and protein synthesis involves tens of enzymatic reactions, many of which are bimolecular, increasing the concentrations and hence proximity of enzymes and other mediating factors should increase the productivity of the system. Evidence supporting this idea was first established by concentrating S30 extract for CFPS reactions, leading to increased protein production rates and yields in both batch and continuous-exchange S30 extract-based CFPS systems (35,36). In iSAT reactions, the total extract protein concentration is roughly 10-fold lower than typical S30 extract-based systems, suggesting opportunity for improving activity by S150 extract concentration.

To assess the impact of S150 extract concentration, we measured the protein synthesis yield in iSAT reactions as a function of final extract protein concentration. The total S150 extract protein concentration was varied from 1.1 to 1.8 mg/ml and changes in volume were offset by addition of S150 extract buffer to maintain consistent salt concentrations. Over this range, iSAT reactions showed a strong upward trend in protein synthesis activity (Supplementary Figure S1), achieving a 5-fold improvement in luciferase production when comparing the highest and lowest extract protein concentrations. Further increases in extract protein concentration, however, were restricted by extract protein aggregation and the need for manipulation of other component volumes within the 15 μl reaction. As a control, we confirmed that the activity of our *in vitro* transcribed rRNA was not the result of unmasking some minor level of contaminating native rRNA from S150 extract or purified TP70; adding TP70 alone did not account for our observed protein synthesis (11). The increase of extract volume required re-optimization of salt and polyamine concentrations, resulting in an additional 50% improvement in iSAT protein synthesis activity (Supplementary Figure S2).

### 3′ modifications of rRNA gene constructs

A key limitation in the original iSAT system was the choice of plasmids encoding 16S and 23S rRNA (pWK1 and pCW1, respectively (31,32)). To produce 16S and 23S rRNA of the proper length, these plasmids need to be linearized prior to *in vitro* transcription (IVT) (31,32). However, our S150 extract contains endonucleases that degrade linear DNA templates, so pWK1 and pCW1 were used in iSAT as circular DNA with no defined 3′ transcriptional termination (11). Without termination, additional 3′ bases are expected on the rRNA, which may interfere with ribosome assembly and activity. Therefore, we introduced modifications at the 3′ end of rRNA genes to assess whether iSAT activity could be increased through improved rRNA processing and transcriptional efficiency.

**Figure 2.**
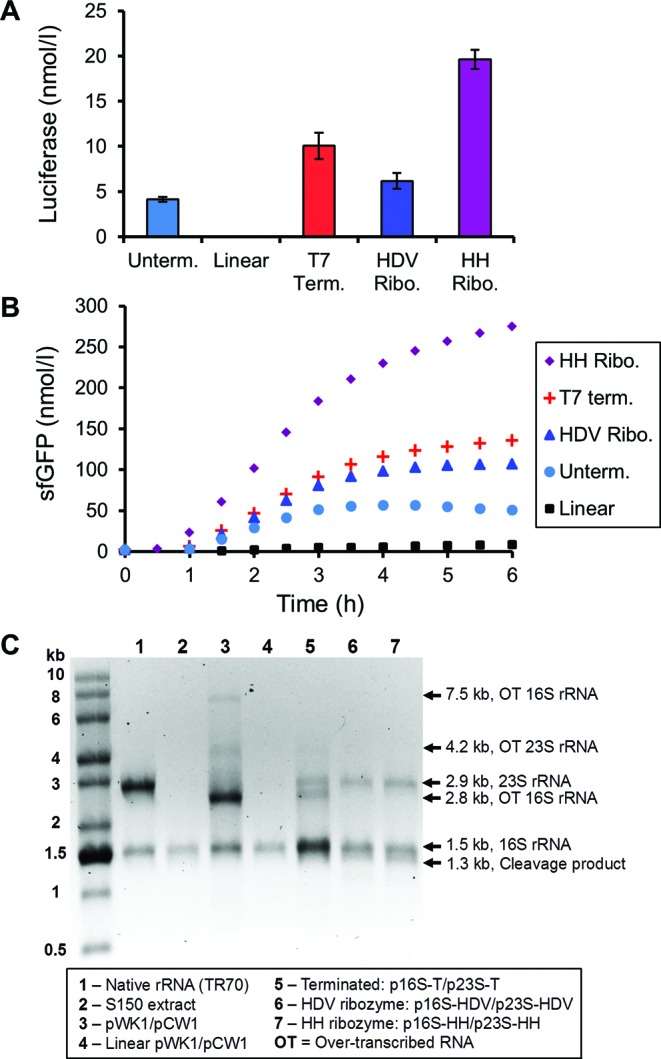
Comparison of iSAT reactions utilizing separate plasmids encoding 16S and 23S rRNA with various 3′ gene modifications. (**A**) Comparison of luciferase production in iSAT reactions utilizing separate 16S and 23S plasmids with 3′ gene modifications shown in Figure 1. Reactions were incubated at 37°C for 4 h. (**B**) Same comparison as (A) for sfGFP production, measured at 30 min intervals from 0 to 6 h while incubating at 37°C. For panels (A) and (B), values show average reporter protein concentrations above background for at least three independent reactions. Error bars in (A) represent standard deviation (s.d.). (**C**) RNA denaturing gel of iSAT reactions utilizing separate 16S and 23S plasmids with various 3′ gene modifications. TR70 control lane contains 1 μg purified *E. coli* rRNA. 16S rRNA in S150 extract control lane indicates presence of residual 16S rRNA in extract. Over-transcribed (‘OT’) bands are RNA bands that represent transcription beyond the intended 16S or 23S rRNA gene and were identified by individual expression of 16S or 23S constructs (Supplementary Figure S3).

We tried several strategies, including: (i) 3′ linearization of pWK1 and pCW1 by the restriction enzymes Bsu36I and AflII, respectively, (ii) termination with T7-TΦ terminators (37), (iii) addition of genes for the self-cleaving hepatitis delta virus (HDV) ribozyme (genomic variant) followed by a T7-TΦ terminator (38) or (iv) addition of genes for the self-cleaving hammerhead (HH) ribozyme (genomic variant from lucerne transient streak virus satellite RNA, satLTSV+) followed by a T7-TΦ terminator (39) (Figure 1). These modifications were introduced for both the 16S and 23S rRNA genes on separate plasmids and iSAT reactions were performed to assess impact on protein synthesis. iSAT reactions were assessed for synthesis of either luciferase after 4 h of incubation (Figure 2A) or sfGFP from 0 to 6 h of incubation (Figure 2B).

As previously observed, linearized plasmids are not viable in iSAT reactions (11). Other modifications, however, showed improvement over the original pWK1 and pCW1 constructs. Addition of T7-TΦ terminators to the 16S and 23S rRNA genes improved both luciferase and sfGFP production by 2.4-fold. Inclusion of 3′ HDV or HH ribozymes also resulted in increased protein production: for HDV, 1.5-fold for luciferase and 1.9-fold for sfGFP, and for HH, 4.8-fold for each reporter protein.

To assess the impact of 3′ modifications on rRNA processing, rRNA purified from iSAT reactions without reporter plasmids were run on a denaturing agarose gel (Figure 2C). Similar reactions were performed with 16S or 23S rRNA plasmids only in order to attribute over-transcribed (OT) RNA bands to 16S or 23S rRNA transcription (Supplementary Figure S3). While 16S and 23S rRNA are expected to run at 1.5 and 2.9 kb, respectively, the original plasmids pWK1 and pCW1 show several OT bands; bands near 2.8 and 7.5 kb are attributed to 16S rRNA over-transcription, and the band at 4.2 kb is attributed to 23S rRNA over-transcription (Figure 2C). Modifications to the plasmids dramatically altered the resulting rRNA. For linearized plasmids, no significant rRNA production beyond contaminating 16S rRNA from the extract was observed on the gel. Meanwhile, T7-TΦ terminated constructs showed a dramatic decrease in OT bands (Figure 2C). Upon addition of the terminators, all three OT bands decreased in intensity, with only the 2.8 kb band still visible. The persistence of the 2.8 kb band is consistent with estimates that the T7-TΦ terminator is 66% efficient (40). Inclusion of 3′ HDV or HH ribozymes eliminated the remaining portion of the 2.8 kb band (Figure 2C). In addition, a 1.3 kb band emerged that likely represents the cleavage product of the 2.8 kb OT band into the correct 16S rRNA size of 1.5 kb. These data suggest that the improved iSAT activity results from the production of rRNA molecules closer to the proper native length.

### Concentration optimization of transcriptional components

An immediate observation from Figure 2C is that 16S and 23S rRNA are not stoichiometrically balanced. Based on band intensities and relative molecular weights, the HH constructs resulted in approximately 2.5 times more 16S rRNA than 23S rRNA on a molar basis. In comparison, native purified rRNA, TR70, showed equimolar quantities as expected (Figure 2C, Lane 1). Further complicating the issue of transcriptional stoichiometry is the need for T7 RNAP to also transcribe reporter protein mRNA. Therefore, we next asked whether iSAT activity could be improved through optimization of plasmid and RNAP concentrations.

**Figure 3.**
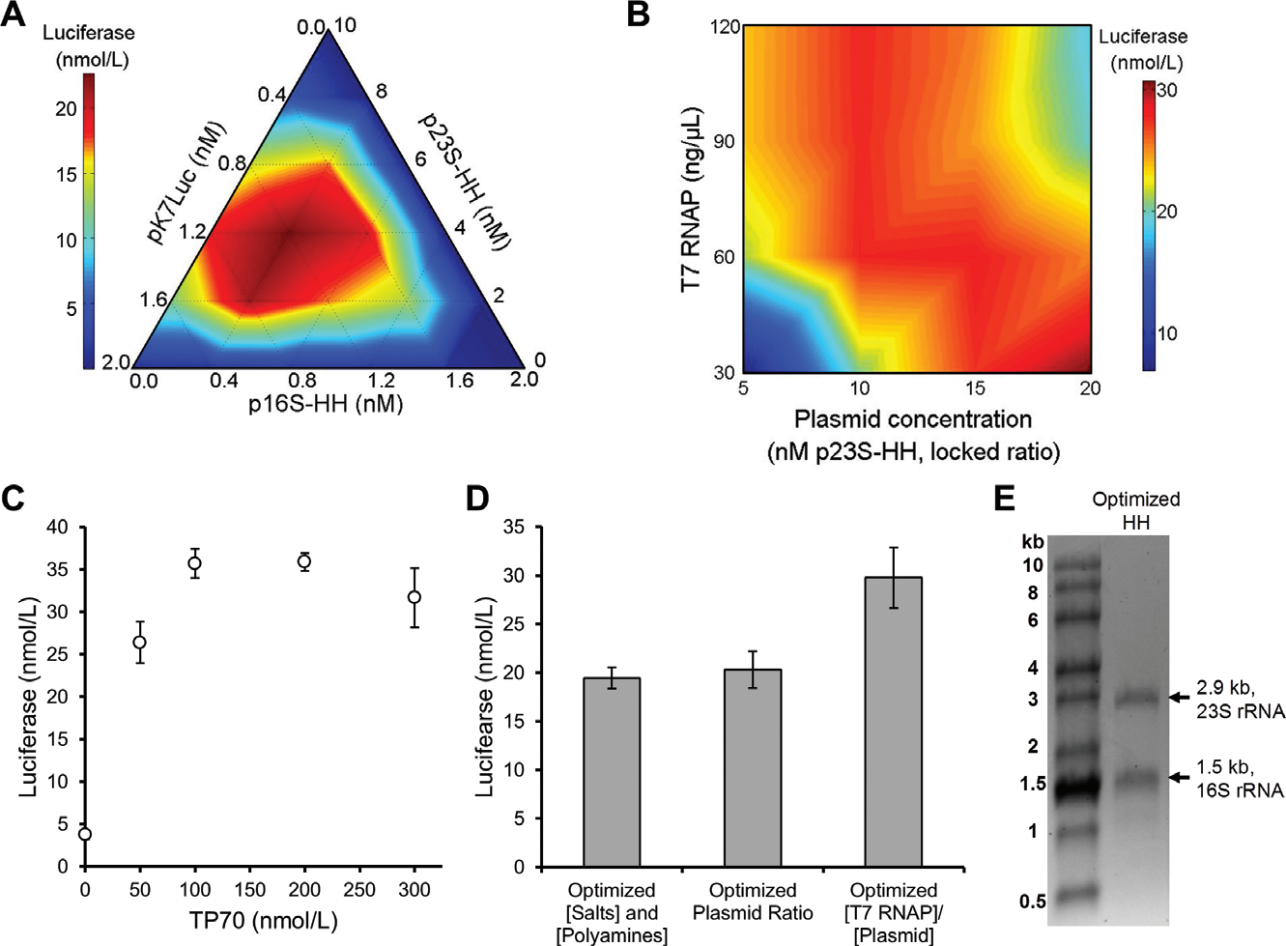
Component optimization for improved iSAT activity from p16S-HH and p23S-HH constructs. (**A**) Ratio optimization of co-transcribed plasmids included in p16S-HH/p23S-HH iSAT reactions for luciferase production. (**B**) Ratio optimization of T7 RNAP and plasmid concentrations included in p16S-HH/p23S-HH iSAT reactions for luciferase production. T7 RNAP was diluted with storage buffer to maintain equivalent salt and buffer concentrations in all reactions. A mix of plasmids was similarly diluted with water. Plasmid concentrations shown are for p23S-HH, with a constant ratio of 2:1:10 for pK7Luc:p16S-HH:p23S-HH, as determined from (A). For panels (A) and (B), values show average luciferase concentrations above background for two independent reactions. (**C**) Effect of TP70 concentration on iSAT activity using p16S-HH and p23S-HH with optimized plasmid and T7 RNAP concentrations. TP70 was diluted with storage buffer to maintain equivalent salt and buffer concentrations in all reactions. (**D**) Summary of improvements in iSAT activity due to plasmid and polymerase concentration optimization. For panels (C) and (D), values show average luciferase concentrations above background with error bars representing s.d. for at least three independent reactions. (**E**) RNA denaturing gel of iSAT reactions utilizing p16S-HH and p23S-HH plasmids and optimized reaction conditions.

Using the HH ribozyme constructs, iSAT reactions were performed with various concentrations of pK7Luc, p16S-HH and p23S-HH in a simplex lattice experimental design (Figure 3A). Based on the need for greater 23S rRNA transcription, p23S-HH concentration was varied from 0 to 10 nM, while pK7Luc and p16S-HH concentrations were only varied from 0 to 2 nM. From this experiment, the molar ratio of pK7Luc to p16S-HH to p23S-HH was set at 2:1:10. The need for excess p23S-HH agrees with the observation that insufficient amounts of 23S rRNA were being transcribed (Figure 2C). Based on these plasmid ratios, total plasmid and T7 RNAP concentrations were varied to determine optimal concentrations for iSAT protein synthesis activity (Figure 3B). Activity was highest for plasmids at a basis of 20 nM p23S-HH (with other plasmids at the set ratio) and 30 ng/μl T7 RNAP, though Figure 3B shows that inversely varying these concentrations also results in high activity.

We next asked whether TP70 concentration could be varied using the improved transcriptional conditions for RNAP and rRNA plasmid concentrations to increase iSAT activity (Figure 3C). While increasing TP70 concentration up to 100 nM increased protein synthesis activity, addition of TP70 beyond 100 nM resulted in visible turbidity and no further increase in protein synthesis (Figure 3C). As S150 and TP70 use similar storage buffers, this result suggests that protein aggregation occurs upon mixing the two protein solutions at high concentrations; it is not possible to assemble more than 100 nM ribosomal equivalents in this iSAT reaction.

Overall, transcriptional tuning of key iSAT components resulted in a 47% improvement in 70S iSAT protein synthesis activity (Figure 3D). However, visualization of the rRNA from an optimized iSAT reaction shows that 16S and 23S rRNA are still not expressed at stoichiometrically balanced levels, as the longer 23S rRNA band should be more intense than an equimolar amount of 16S rRNA (Figure 3E).

### Use of T7-promoted native rRNA operon in iSAT reactions

**Figure 4.**
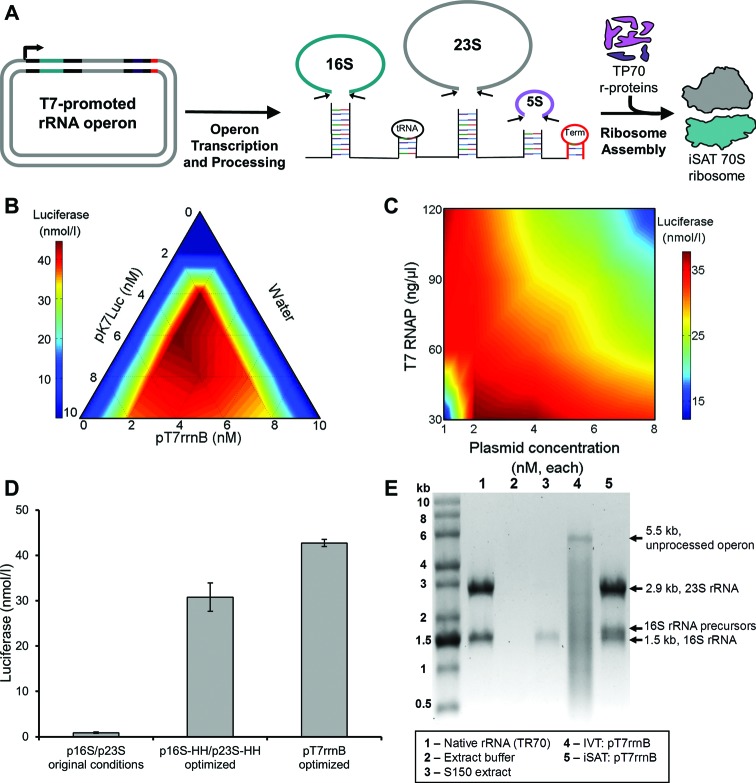
Component optimization for iSAT system utilizing T7-promoted rrnB operon. (**A**) Diagram of expected transcription and processing of rrnB operon encoding 16S, 23S and 5S rRNA from a T7-promoted plasmid. Expected processing of transcribed RNA includes cleavage of rRNA by RNases present in S150 extract to allow for 70S ribosome assembly. All steps occur in isothermal iSAT reactions (37°C), and resulting ribosomes are assessed for activity by measuring reporter protein production, as depicted in Figure 1. (**B**) Ratio optimization of co-transcribed plasmids included in operon-based iSAT reactions for luciferase production. (**C**) Ratio optimization of T7 RNAP and plasmid concentration in operon-based iSAT reactions for luciferase production. T7 RNAP was diluted with storage buffer to maintain equivalent salt and buffer concentrations in all reactions. The plasmids pK7Luc and pT7rrnB were mixed at an equimolar ratio as determined from (B). For panels (B) and (C), values show average luciferase concentrations above background for at least two independent reactions. (**D**) Comparison of luciferase production for iSAT reactions utilizing p16S/p23S, p16S-HH/p23S-HH or pT7rrnB. Values show average luciferase concentrations above background with error bars representing s.d. for at least three independent reactions. (**E**) RNA denaturing gel of iSAT reactions utilizing pT7rrnB. The *in vitro* transcribed (IVT) reaction (Lane 4) included all components of iSAT reactions except S150 extract, which was replaced with extract buffer to maintain salt concentrations. TR70 control lane includes 1 μg RNA.

To achieve stoichiometric balance of rRNA transcription, we looked to nature for inspiration. *In vivo* ribosome biogenesis utilizes co-transcription of operons encoding 5S, 16S and 23S rRNA. However, use of an operon in iSAT reactions would depend on the correct folding of intergenic regions into stem loops and the presence and activity of RNases required for processing of the operon into individual rRNA molecules (5,6) (Figure 4A). We asked whether a native rRNA operon could be transcribed and assembled into active ribosomes in the iSAT system, hypothesizing that enzymes in the crude S150 extract could process *in vitro* transcribed RNA of the operon. We placed the rrnB rRNA operon behind a T7 promoter and the resulting construct, pT7rrnB, was used in place of separate plasmids encoding 16S and 23S rRNA.

Despite previous reports that plasmid-borne rRNA from a T7-promoted rRNA operon at 37°C leads to hardly any assembled and active 70S ribosomes *in vivo* (10), our initial experiments showed that using a T7-promoted rRNA operon in the iSAT system resulted in functional ribosomes. Therefore, we carried out a series of optimization reactions to explore the effect of transcriptional component concentrations on the operon-based iSAT system. First, optimization of the plasmid ratio showed a 1:1 molar ratio of pK7Luc to pT7rrnB resulted in maximal activity (Figure 4B). Next, optimization of plasmid and T7 RNAP concentrations (Figure 4C) showed a trend similar to that seen for the separate plasmid system (Figure 3B); inversely varying T7 RNAP and plasmid concentrations resulted in the highest activities. Based on these data, for maximal protein synthesis, plasmid concentrations were set at 4 nM for each plasmid and 30 ng/μl for T7 RNAP. Finally, varying the TP70 concentration showed no significant increase in operon-based iSAT activity beyond 100 nM TP70 (Supplementary Figure S4), also similar to the separate plasmid iSAT system. Overall, the operon-based system showed a 39% increase in luciferase synthesis over the optimized separate plasmid system, and a total 45-fold improvement over the original iSAT conditions (Figure 4D).

Since iSAT reactions using the pT7rrnB operon plasmid resulted in active protein synthesis, we hypothesized that the RNA was correctly processed into individual rRNA molecules. To test this hypothesis, iSAT reactions were incubated without reporter plasmid for visualization of the transcribed rRNA. As a control for RNA self-cleavage, an IVT reaction using S150 extract buffer in place of S150 extract was performed to determine if extract proteins (e.g. RNA processing enzymes) were responsible for any observed RNA processing. Total RNA of each reaction was purified and run on a denaturing agarose gel (Figure 4E). RNA of the IVT reaction appears at approximately 5.5 kb, the size of the complete, unprocessed operon. In contrast, the operon-based iSAT reaction shows strong bands at the correct 16S and 23S rRNA sizes, though smearing larger than the 16S band may indicate incomplete processing of 16S rRNA precursors. Additionally, the ratio of 16S and 23S rRNA in the operon-based iSAT reaction closely matches the ratio of the control rRNA bands; quantification of band intensity shows that the operon-based iSAT system generates approximately equimolar amounts of 16S and 23S rRNA.

**Figure 5.**
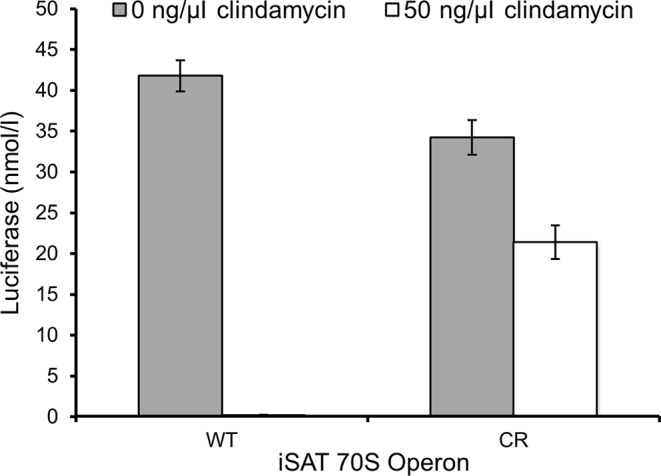
Demonstration of ribosome modification in operon-based iSAT system. The point mutation A2058U of the 23S rRNA gene was made to pT7rrnB (WT) to confer resistance to the antibiotic clindamycin, and the resulting plasmid, pT7rrnB-CR (CR), was used in iSAT reactions. Clindamycin was added to the appropriate iSAT reactions at a final concentration of 50 ng/μl prior to incubation, and activity was assessed through luciferase production at 37°C for 4 h. WT iSAT reactions showed an average luciferase concentration of less than 0.25 nM in the presence of clindamycin. Values show average luciferase concentrations above background with error bars representing s.d. for three independent reactions.

Since the ability to manipulate rRNA sequence, particularly that of the 23S rRNA, is a key feature of the iSAT system, we wished to demonstrate the incorporation of unique functionality into ribosomes generated from the operon. As previously reported, iSAT technology utilizing the separate plasmid system can be used to generate ribosomes that are resistant to the antibiotic clindamycin via a 23S rRNA point mutation, A2058U (11). The same approach was applied here by introducing the A2058U mutation into the 23S rRNA gene of pT7rrnB. The construct conferring clindamycin resistance, pT7rrnB-CR, was used in iSAT reactions with and without clindamycin. At 50 ng/μl clindamycin, ribosomes derived from transcription of pT7rrnB-CR retained 51% activity, whereas ribosomes derived from transcription of pT7rrnB retained 0.5% activity, as defined by batch luciferase synthesis yields (Figure 5). This result demonstrates the potential for constructing mutant ribosomes directly from rRNA operon plasmid DNA in a one-step reaction, streamlining the process for *in vitro* ribosome construction and study.

### Comparison of 70S iSAT ribosomes to assembled or purified 70S ribosomes

**Figure 6.**
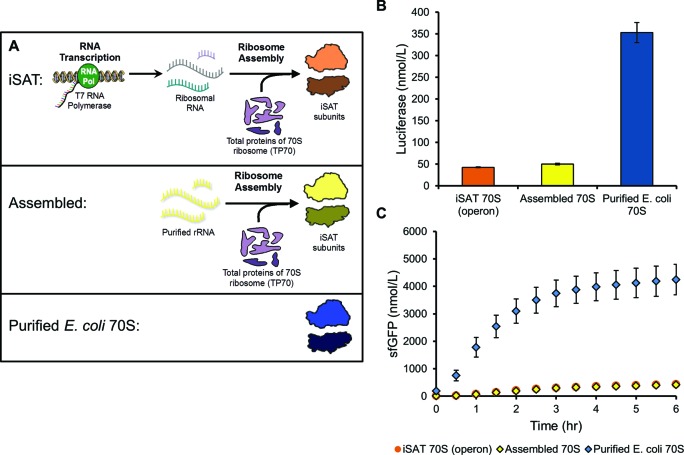
Comparison of operon-based iSAT ribosomes, assembled 70S ribosomes, and purified 70S ribosomes for *in vitro* protein translation. (**A**) Diagram showing the difference between iSAT ribosomes, assembled ribosomes, and purified *E. coli* 70S ribosomes. iSAT ribosomes are made from rRNA transcribed in the iSAT reaction along with purified r-proteins. Assembled ribosomes are made from purified native rRNA and purified r-proteins. Purified *E. coli* 70S ribosomes are cell-derived and added intact to cell-free TX-TL reactions. (**B**) Comparison of luciferase production for operon-based iSAT ribosomes, *in vitro* assembled ribosomes, or purified 70S ribosomes in 15 μl cell-free reactions incubated for 4 h at 37°C. Each condition used a maximum of 100 nM ribosomes, as concentrations of iSAT and assembled ribosomes were limited by use of 100 nM TP70. For assembled and purified ribosomes, pT7rrnB was included at optimized conditions to maintain an equivalent transcriptional load on T7 RNA polymerase; pilot studies suggest that rRNA transcription had no effect on assembled or purified ribosomes. (**C**) Same comparison as (B) for sfGFP production, measured at 30 min intervals from 0 to 6 h while incubating at 37°C. Note that data for iSAT and assembled ribosomes result in overlapping data points. For panels (B) and (C), values show average reporter protein concentrations above background with error bars representing s.d. for three independent reactions.

**Figure 7.**
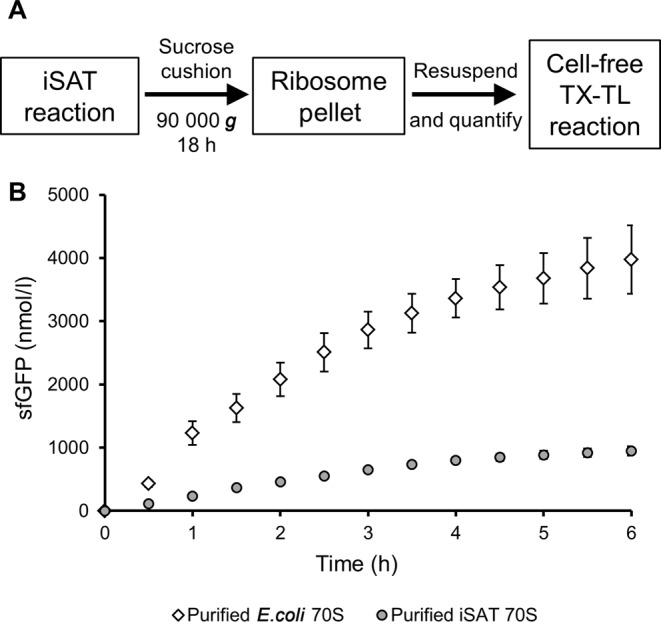
Purification of *in vitro-*constructed iSAT 70S ribosomes for comparison to purified *E. coli* 70S ribosomes. (**A**) Schematic of iSAT 70S ribosome purification. iSAT ribosomes were subjected to ultracentrifugation at 90 000 *g* for 18 h. The supernatant was removed and the clear ribosome pellet was resuspended in a HEPES-based high salt buffer. Purified iSAT ribosomes were then used in cell-free transcription and translation (TX-TL) reactions. (**B**) Comparison of sfGFP production by purified iSAT 70S ribosomes and purified *E. coli* 70S ribosomes. Ribosomes were added to S150 extract-based cell-free TX-TL reactions at a final concentration of 100 nM. Reactions were incubated at 37°C and green fluorescence was measured at 30 min intervals from 0 to 6 h. Values show average sfGFP concentrations above background with error bars representing s.d. for three independent reactions.

Activity of ribosomes created in iSAT reactions depends on both transcriptional and assembly processes, as well as protein synthesis. To separate the current limitations associated with each process, protein synthesis activities were compared for operon-based iSAT 70S ribosomes, 70S ribosomes assembled in S150 extract from purified rRNA and purified r-proteins (‘assembled 70S’) and purified, intact 70S ribosomes (Figure 6A). To maintain equivalent transcriptional load on the T7 RNAP, pT7rrnB was included in all reactions, as pilot studies showed no effect of excess transcribed rRNA on assembled or purified ribosome activity (data not shown). Protein synthesis by iSAT ribosomes relative to assembled ribosomes was 83% and 105% for luciferase and sfGFP, respectively (Figure 6B and C). On the other hand, purified 70S ribosomes resulted in greater luciferase and sfGFP synthesis than iSAT ribosomes (Figure 6B and C). However, it was difficult to directly compare these reactions because reactions with purified 70S ribosomes do not have a lag time associated with rRNA synthesis and ribosome assembly. In addition, while iSAT ribosome assembly was capped at 100 nM by the TP70 concentration, the true concentrations of iSAT ribosomes in the reactions were unknown. Therefore, we purified iSAT-assembled 70S ribosomes directly from iSAT reactions through ultracentrifugation on a sucrose cushion, resuspension in a HEPES-based high salt buffer, and quantification from A_260_ readings (Figure 7A). Purified iSAT ribosomes were subsequently added to a cell-free transcription and translation reaction for synthesis of sfGFP. This allowed for the direct comparison of the translational activity of 100 nM each of iSAT 70S ribosomes versus purified *E. coli* 70S ribosomes (Figure 7B). After a 6 h batch reaction at 37°C, we observed that iSAT-assembled ribosomes have about 20% the activity of purified native ribosomes.

## DISCUSSION

iSAT provides a powerful new tool for observation and study of ribosome biogenesis under more physiological conditions than conventional reconstitution (11). Specifically, the system enables rRNA transcription to be integrated with ribosome assembly and protein synthesis. Here, we demonstrated that transcriptional tuning is critical for iSAT, with advances producing a 45-fold increase in 70S iSAT activity relative to previous results (11). Importantly, iSAT ribosomes now display ∼85–100% protein synthesis activity compared to ribosomes assembled from purified native rRNA. This contrasts with conventional *E. coli* ribosome reconstitution protocols, whereby 50S subunits assembled from *in vitro* transcripts of *E. coli* 23S rRNA have ∼0.02–3% the activity of those assembled from mature rRNA (8,25). Thus, the iSAT platform provides significant efficiency advantages as compared to previous works, noting that the measure of functional activity in iSAT reactions is the synthesis of full-length, easy to monitor reporter proteins.

Transcriptional tuning of iSAT reactions resulted in improvements to protein synthesis activity and enhanced our understanding of the system. First, we demonstrated that a synthetic approach, the introduction of ribozyme sequences to the 3′ end of rRNA gene constructs, improved iSAT activity 5-fold by processing rRNA and reducing the presence of over-transcribed RNA bands. However, our data indicated that we were not producing stoichiometric amounts of rRNA as occurs for *in vivo* ribosome biogenesis. Thus, we further improved iSAT activity by adjusting plasmid and RNAP concentrations. Because the plasmid concentrations were interdependent, we applied a simplex lattice experimental design, observing that the optimal ratio of 23S to 16S rRNA plasmid was 10:1. These data implied that the assembly of 50S subunits limited the construction of 70S ribosomes in iSAT.

Modification of rRNA-encoding plasmids and optimization of component concentrations improved iSAT activity, and further highlighted the need to better mimic rRNA synthesis as it occurs *in vivo.* We therefore turned to the native rRNA operon to further improve *in vitro* ribosome construction through stoichiometric expression of rRNA molecules and utilization of native rRNA processing mechanisms. RNA gel analysis of operon-based iSAT reactions clearly shows more efficient and stoichiometrically balanced rRNA transcription than the previous iSAT system. We observed more efficient 23S rRNA synthesis and processing from less plasmid when using the T7-promoted rrnB operon, suggesting that strong determinants for ordered assembly are found in the various operon sequence components. That said, the presence of longer 16S rRNA precursors suggests incomplete processing by RNases in the S150 extract, hinting that effective processing of all rRNA may further improve iSAT activity. One cause of incomplete processing may be the use of an RNase inhibitor cocktail in the preparation of S150 extract, which may affect the activity of RNases required for operon processing (5,6). Alternatively, the use of T7 polymerase may cause transcription to occur too quickly for correct folding or processing of all rRNA molecules.

We were surprised that the T7-promoted rRNA operon construct worked so well *in vitro* at 37°C. This result could imply that the rRNA transcription rate by T7 RNAP and the corresponding ribosome assembly rate are more similar in the iSAT system than what occurs *in vivo*, which allows for *in vitro* construction of functional 70S ribosomes at 37°C. As a follow-on experiment, we prepared iSAT reactions using an rrnB operon behind the strong leftward promoter (*P*_*L*_) of bacteriophage lambda; the *P_L_*-promoter constitutively utilizes native *E. coli* RNAP (41). We showed that this construct also resulted in iSAT protein synthesis activity, even without addition of *E. coli* RNAP (Supplementary Figure S5). Importantly, this suggests that the native RNAPs are present and active in S150 extract. These experiments further support the ability of S150 extract proteins to facilitate *in vitro* ribosome construction. Additionally, we demonstrated that the operon-based iSAT system was susceptible to rRNA sequence modification by creating clindamycin-resistant ribosomes through a point mutation of the operon, as previously shown for the separate plasmid iSAT system (11).

Figure 7 shows that the overall protein synthesis activity of iSAT ribosomes is now about 20% that of purified 70S ribosomes. Even though this opens the way to using iSAT for studying ribosome synthesis, there are still some exciting opportunities to further improve the efficiency of iSAT and characterize generated ribosomes. First, differences in iSAT-assembled ribosomes and purified *E. coli* ribosomes may impact activity. Thus, there is a need to characterize the molecular and functional components of iSAT ribosomes. We are interested to know, for example, if the rRNA is being post-transcriptionally modified in the lysate. The status of the rRNA modifications is currently unknown, and a focal point of future work. A new method for analyzing rRNA modifications by isotope labeling and mass spectrometry was recently published by Popova and Williamson (42), and such a method coupled with the iSAT technology could provide new insight into ribosome biogenesis. Second, we hypothesize that substrate instabilities, typically present in cell-free transcription and translation systems (13), could limit iSAT reactions. In subsequent work, we are exploring the causes of reaction termination in iSAT to improve activity and protein yields. Third, TP70 and S150 protein stability and concentration impact assembly efficiency (Figure 3C, Supplementary Figures S1 and S4). TP70 concentration caps iSAT ribosome concentration at 100 nM, while *in vivo* ribosome concentrations range from approximately 10 to 100 μM (43). Additionally, S150 extract protein concentration, now at 1.8 mg/ml, is still significantly more dilute than *E. coli* cytoplasmic conditions (34). Achieving protein concentrations in iSAT reactions closer to *in vivo* concentrations will be a critical next step toward fully replicating ribosome biogenesis *in vitro*. Even though there are opportunities to improve iSAT technology, the 45-fold improvement in iSAT protein synthesis presented in this work is a significant advancement that increases the potential applications of this technology.

In summary, the advances to iSAT reported here will enable future studies to tease apart functions of individual ribosomal components and steps in ribosome biogenesis processes, and ultimately in the ability to make modified ribosomal variants with new functionalities. *In vivo* ribosome studies are limited by cell viability constraints and transformation efficiencies (7). The iSAT technology should overcome these restrictions, allowing for in-depth understanding and manipulation of complex 70S ribosomes. For example, the demonstration of rRNA processing in iSAT suggests that this approach can be used to explore the systems-level impact of positive and negative effectors of ribosome biogenesis. Currently, over 20 *E. coli* proteins have been identified as ribosomal assembly cofactors, including helicases, GTPases and chaperones, which facilitate RNA folding and RNA–protein interactions in the formation of the 30S and 50S subunits (6,44). iSAT can provide an alternative approach to assess the impact of these factors on wild-type and mutant ribosomes by modulating the extract source strain (e.g. deletion mutants, etc.). Looking forward, we anticipate that iSAT will contribute meaningfully towards efforts to completely synthesize ribosomes *in vitro* and provide new opportunities to engineering ribosomes with altered properties.

## SUPPLEMENTARY DATA

Supplementary Data are available at NAR Online.

SUPPLEMENTARY DATA
