# Supplementary material for: The impact of transcriptional tuning on in vitro integrated rRNA transcription and ribosome construction
Source: Nucleic Acids Res. 2014 May 3;42(10):6774–85. doi: 10.1093/nar/gku307 (PMC4041470; doi:10.1093/nar/gku307)
Supplement: SUPPLEMENTARY DATA [file supp_42_10_6774__index.html]

The impact of transcriptional tuning on in vitro integrated rRNA transcription and ribosome construction — The impact of transcriptional tuning on in vitro integrated rRNA transcription and ribosome construction — SUPPLEMENTARY DATA 

# The impact of transcriptional tuning on *in vitro* integrated rRNA transcription and ribosome construction

## SUPPLEMENTARY DATA

**Files in this Data Supplement:**

- SUPPLEMENTARY DATA
