# Supplementary material for: The impact of transcriptional tuning on in vitro integrated rRNA transcription and ribosome construction
Source: Nucleic Acids Res. 2014 May 3;42(10):6774–85. doi: 10.1093/nar/gku307 (PMC4041470; doi:10.1093/nar/gku307)
Supplement: SUPPLEMENTARY DATA [file supp_gku307_nar-00445-r-2014-File010.pdf]

The impact of transcriptional tuning on *in vitro* integrated rRNA transcription and ribosome construction

Brian R. Fritz[1] and Michael C. Jewett[1,2,3,4,5,*]

[1] Department of Chemical and Biological Engineering
[2] Interdepartmental Program in Biological Sciences,
[3] Northwestern Institute on Complex Systems,
[4] Institute for Bionanotechnology in Medicine,
[5] Chemistry of Life Processes Institute,

Northwestern University, 2145 Sheridan Road, Evanston, IL 60208, USA.

* To whom correspondence should be addressed. Tel: +1 847 467 5007; Email: m-jewett@northwestern.edu

Supplementary Information

Submitted to *Nucleic Acids Research*

Supplementary Table S1

Construct	Source plasmid	Forward primer (5'-3')	Reverse primer (5'-3')
p16S-T	pWK1 (16S rRNA)	**AAACGGGTCTTGAGGGGTTTTTTG**TCTAGAGTCGACCTGCAGG	**AGAGGCCCCAAGGGGTTATGCTAG**CCTAAGGAGGTGATCCAACC
p23S-T	pCW1 (23S rRNA)	**AAACGGGTCTTGAGGGGTTTTTTG**AAGCTGCAGGCATGC	**AGAGGCCCCAAGGGGTTATGCTAG**AAGGTTAAGCCTCACGGT
p16S-HDV	p16S-T	**TTCCGAGGGGACCGTCCCCTCGGTAATGGCGAATGGGACCCA**CTAGCATAACCCCTTGGGG	**TGTTGCCCAGCCGGCGCCAGCGAGGAGGCTGGGACCATGCCGGCCA**CCTAAGGAGGTGATCCAACC
p23S-HDV	p23S-T	**TTCCGAGGGGACCGTCCCCTCGGTAATGGCGAATGGGACCCA**CTAGCATAACCCCTTGGGG	**TGTTGCCCAGCCGGCGCCAGCGAGGAGGCTGGGACCATGCCGGCCA**CTTAAGGTTAAGCCTCACGGT
p16S-HH	p16S-T	**CCCGGTAGGGCCGAAACCTA**CTAGCATAACCCCTTGGGG	**CCATCTTCAGTGAGCGGGTATCACGCTCAGA**CCTAAGGAGGTGATCCAACC
p23S-HH	p23S-T	**ATGGCCCGGTAGGGCCGAAACTTA**CTAGCATAACCCCTTGGG	**CTTCAGTGAGCGGGTATCACGCTCAGA**CTTAAGGTTAAGCCTCACGG
pT7rrnB	pAM552A	GGCCGCTGAGAAAAAGC	**CCTATAGTGAGTCGTATT**AACCAGCAAAAGGCCAGG

Supplementary Table S1. Primers used for plasmid modifications through phosphorylation, inverse PCR and blunt end ligation. Bold sequences indicate additions to the source plasmid.

Supplementary Table S2

Reagents		Reagent concentration range	Concentrations for separate plasmid iSAT reactions	Concentrations for operon-based iSAT reactions
**Salts (in addition to component buffers):**				
Magnesium glutamate ($Mg(Glu)_2$)		0 - 15 mM	7.5 mM	7.5 mM
Ammonium glutamate ($NH_4(Glu)$)		0 - 25 mM	0 mM	0 mM
Potassium glutamate (KGlu)		0 - 500 mM	167 mM	167 mM
**Polyamines (in addition to component buffers):**				
Spermidine		0.0 - 5.0 mM	1.5 mM	1.5 mM
Putrescine		0.0 - 5.0 mM	1.0 mM	1.0 mM
**Transcriptional master mix, consisting of:**				
ATP		1.20 mM	1.20 mM	1.20 mM
GTP		0.85 mM	0.85 mM	0.85 mM
UTP		0.85 mM	0.85 mM	0.85 mM
CTP		0.85 mM	0.85 mM	0.85 mM
Folinic acid		34.0 µg/mL	34.0 µg/mL	34.0 µg/mL
tRNA		171 µg/mL	171 µg/mL	171 µg/mL
**Transcriptional and translational components:**				
rRNA plasmid(s):	p16S construct	0 - 4 nM each	2.0 nM	-
	p23S construct	0 - 20 nM each	20.0 nM	-
	pT7rrnB or pAM552A	1 - 10 nM each	-	4.0 nM
Reporter plasmid:	pK7Luc or pY71sfGFP	0 - 10 nM	4.0 nM	4.0 nM
T7 RNA polymerase		30 - 120 µg/mL	30 µg/mL	30 µg/mL
Purified 70S ribosomes		100 nM	100 nM	100 nM
Total protein of 70S ribosomes (TP70)		0 - 300 nM	200 nM	200 nM
Total rRNA of 70S ribosomes (TR70)		100 nM	100 nM	100 nM
**Other components - substrates, cofactors, buffers:**				
20 amino acids		2.00 mM	2.00 mM	2.00 mM
NAD		0.33 mM	0.33 mM	0.33 mM
CoA		0.27 mM	0.27 mM	0.27 mM
HEPES-KOH, pH 7.6		57.00 mM	57.00 mM	57.00 mM
Oxalic acid		4.00 mM	4.00 mM	4.00 mM
PEP		42.00 mM	42.00 mM	42.00 mM

Supplementary Table S2. Reagents and concentrations used in iSAT reactions.

Supplementary Figure S1

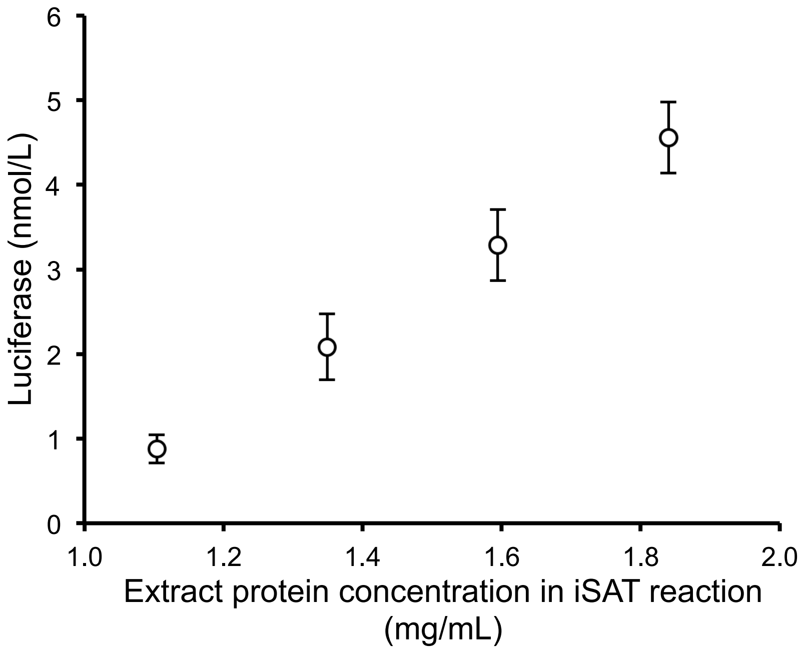

Supplementary Figure S1. The impact of S150 extract protein concentration on iSAT activity. iSAT reactions were performed with different concentrations of S150 extract using the pWK1 and pCW1 plasmids encoding 16S and 23S rRNA, respectively. S150 extract buffer was used to offset differences in extract volumes to maintain equal salt and buffer concentrations in all reactions. iSAT reactions without rRNA plasmids were performed to establish background expression levels. Values show average luciferase concentrations above background with error bars representing standard deviation (s.d.) for 3 independent reactions.

Supplementary Figure S2

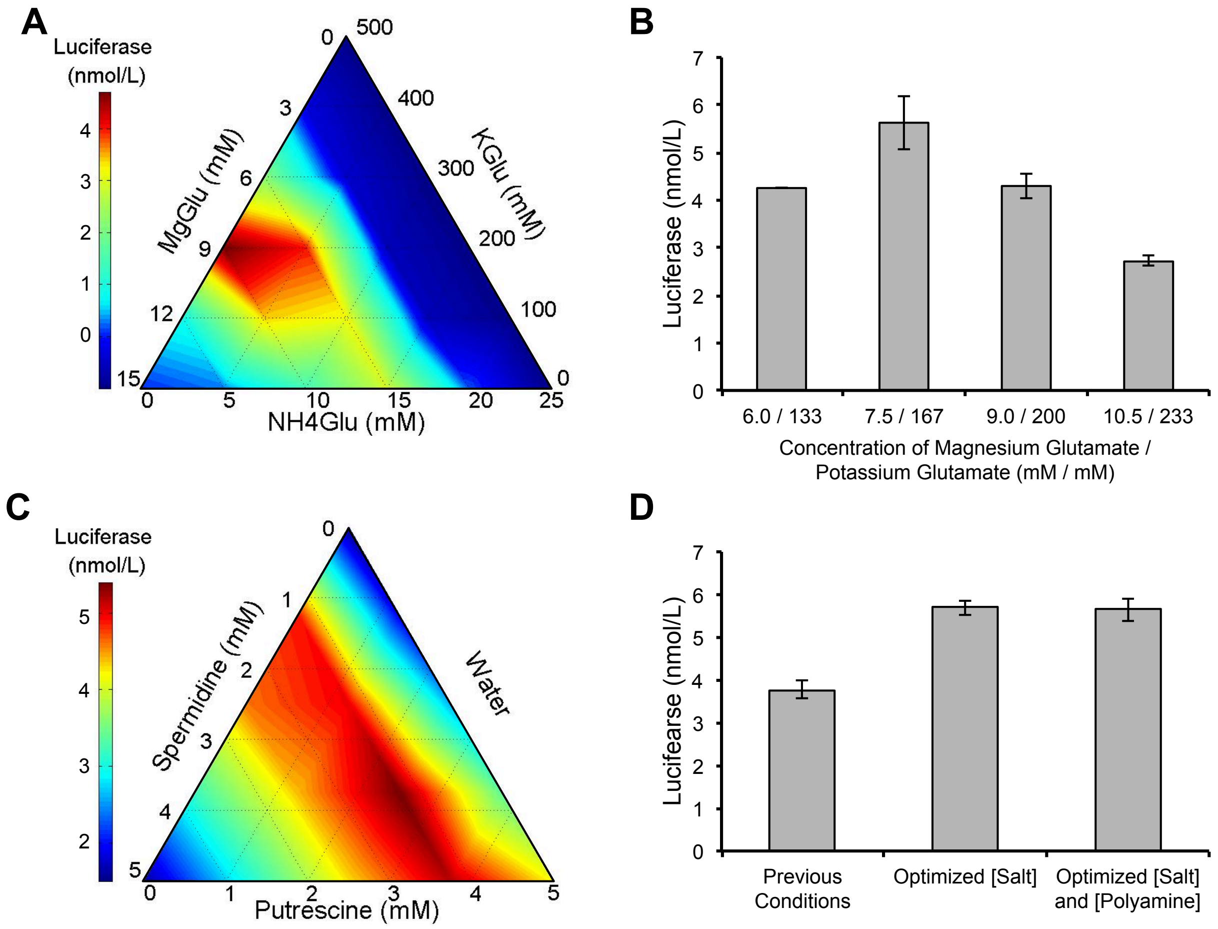

Supplementary Figure S2. Salt and polyamine concentration optimization for separate plasmid iSAT reactions with increased extract protein concentration. Reaction conditions are as shown in Supplementary Table 1 using 1.8 mg/mL S150 extract proteins (**Supplementary Figure S1**). (A) Ratio optimization of magnesium glutamate (MgGlu), potassium glutamate (KGlu) and ammonium glutamate (NH_4Glu) in iSAT reactions. Salt concentrations listed neglect salts included in S150 extract, TP70, and T7 RNA polymerase buffers. (B) Optimization of salt concentrations in iSAT reactions based on ratio of MgGlu and KGlu determined in (A). NH_4Glu was excluded as it was determined to be detrimental to iSAT activity beyond the amounts introduced in component buffers. Final salt concentrations were set at 7.5 mM MgGlu and 167 mM KGlu in addition to salts in the protein and rRNA storage buffers. (C) Ratio and concentration optimization of the polyamines spermidine and putrescine in iSAT reactions. Optimized salt concentrations were used (A, B). Final polyamine concentrations were set at 1.5 mM spermidine and 1.0 mM putrescine in addition to polyamines in the protein storage buffers. (D) Summary of improvements in iSAT activity due to salt optimization (A, B) and polyamine optimization (C). For panels (A) and (C), values show average luciferase concentrations above background for two independent reactions. For panels (B) and (D), values show average luciferase concentrations above background with error bars representing s.d. for 3 independent reactions.

Supplementary Figure S3

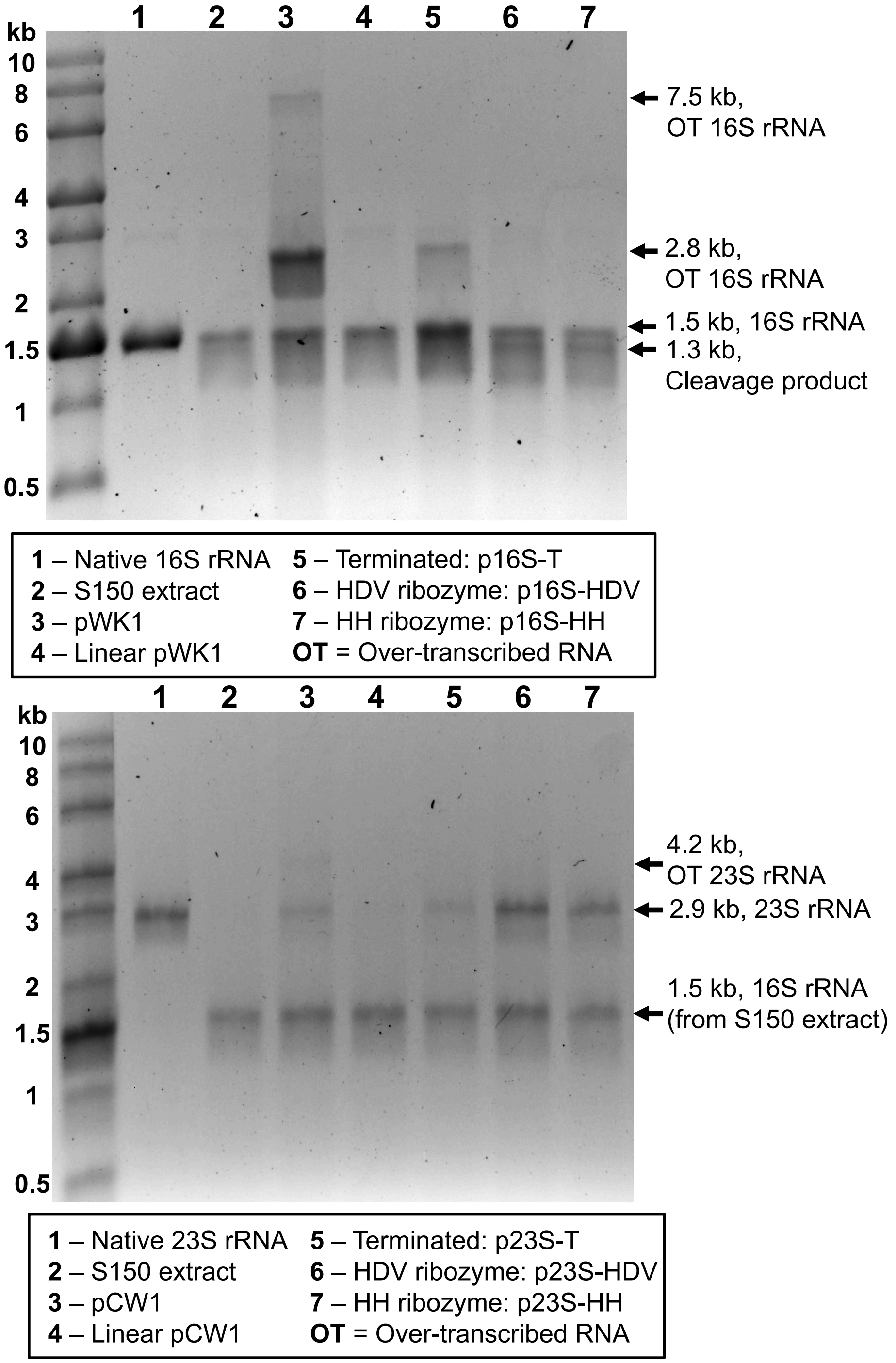

Supplementary Figure S3. RNA denaturing gels of transcription from rRNA constructs with various 3' gene modifications in iSAT reactions. (A) RNA denaturing gel of iSAT reactions expressing 16S rRNA plasmids with 3' gene modifications. Native 16S rRNA lane contains 350 ng RNA. (B) RNA denaturing gel of iSAT reactions expressing 23S rRNA plasmids with 3' gene modifications. Native 23S rRNA lane contains 500 ng RNA. Presence of 16S rRNA bands indicates residual 16S rRNA in S150 extract.

Supplementary Figure S4

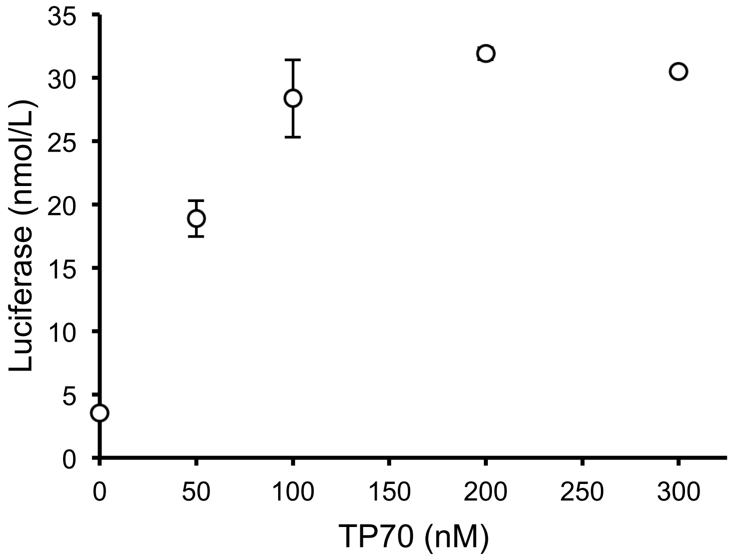

Supplementary Figure S4. Effect of TP70 concentration on operon-based iSAT activity with optimized plasmid and T7 RNAP concentrations (**Figure 4, Supplementary Table S2**). TP70 was diluted with storage buffer to maintain equivalent salt and buffer concentrations in all reactions. Values show average luciferase concentrations above background with error bars representing s.d. for 3 independent reactions.

Supplementary Figure S5

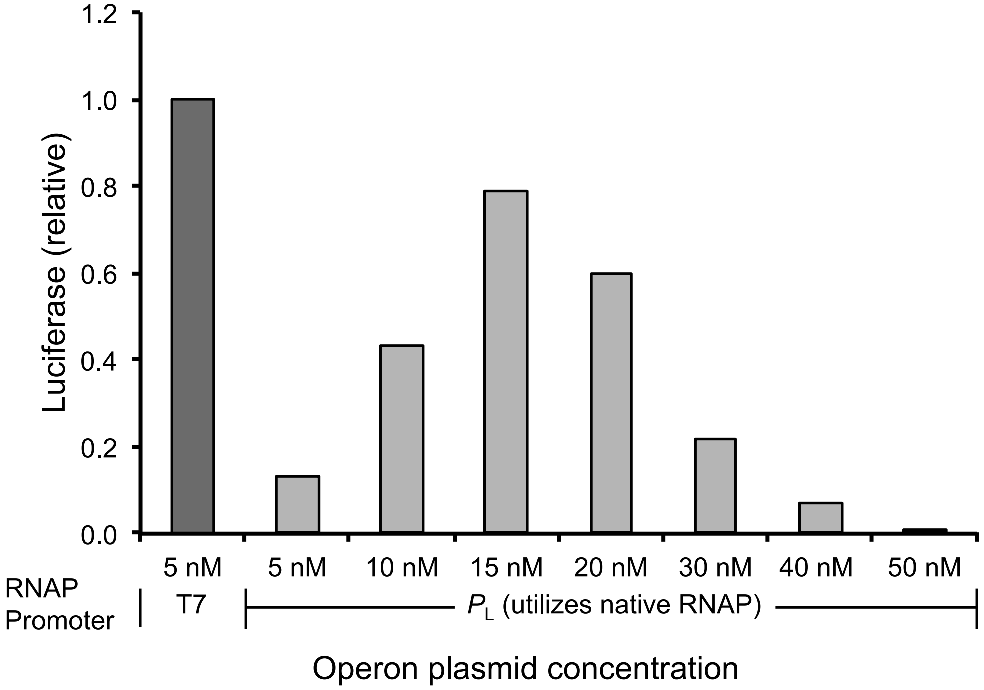

Supplementary Figure S5. Demonstration of iSAT reactions utilizing rRNA operon genes in place of separate plasmids encoding 16S or 23S rRNA. The rRNA operon rrnB was located behind either the T7 promoter (pT7rrnB) or the P_L promoter (pAM552A). Activity of iSAT reactions using the P_L promoter suggests that native *E. coli* RNA polymerase (RNAP) is present and active in the S150 extract. Protein synthesis activity of iSAT reactions is shown as relative luciferase production. All reactions included 30 ng/µL T7 RNAP and 5 nM pK7Luc (T7-promoted).
